# Facilitators and barriers to the uptake of COVID-19 vaccine precaution dose among adult population: qualitative analysis across six different states of India

**DOI:** 10.3389/fpubh.2023.1293600

**Published:** 2024-01-24

**Authors:** Sheela Godbole, Vishal Diwan, Saibal Das, Mahendra M. Reddy, Madhanraj Kalyanasundaram, Dilipkumar Rajendran, Dipankar Biswas, Megha Mamulwar, Rajnarayan R. Tiwari, Joydeep Majumder, Ayush Mishra, Santhosh Kumar Muthusamy, M. Memeenao, Nivedita Gupta, Swati Gupta, Leyanna Susan George, Prajakta Kadale, Tanwi Trushna, Indranil Saha, Umaer Alam, Jeyashree Kathiresan, Sanjib Kumar Phukan, Suvarna Sane, Kalyan Bhowmik, Girijesh Kumar Yadav, Vinaykumar Krishnamurthy, Punananda Gogoi, Kedar Padhye, Rajni Kant, Pramit Ghosh, Mahesh Kharat, Aditi Kulkarni

**Affiliations:** ^1^ICMR-National AIDS Research Institute, Pune, India; ^2^ICMR-National Institute for Research in Environmental Health, Bhopal, India; ^3^ICMR-Centre for Ageing and Mental Health, Kolkata, India; ^4^ICMR-Regional Medical Research Centre Gorakhpur, Gorakhpur, India; ^5^ICMR-National Institute of Epidemiology, Chennai, India; ^6^ICMR-Regional Medical Research Centre, NE Region, Dibrugarh, India; ^7^Division of Communicable Diseases, Indian Council of Medical Research, New Delhi, India; ^8^Directorate of Public Health and Preventive Medicine, Government of Tamil Nadu, Chennai, India

**Keywords:** facilitators, barriers, COVID-19, precaution dose, vaccine hesitancy

## Abstract

**Introduction:**

India launched the COVID-19 vaccination drive on 16th January 2021 by vaccinating the adult population above 18 years of age. This was followed by the introduction of an additional precaution dose. As on 18th October 2022, 1,02,66,96,808 (1.02 Billion) first dose and 94, 95, 39,516 (949 Million) second doses of COVID-19 vaccine were administered. However, when compared to the uptake of the primary doses, the precaution dose uptake lagged behind with only 21,75, 12,721 (217 million) doses administered. Even though, the uptake of the primary doses remained optimal, irrespective of different interventions by the Government of India, the uptake of the precaution dose remained poor. In this context, the Ministry of Health & Family Welfare wanted to understand the facilitators and Barriers for precaution dose uptake among adults so that future immunization campaigns could address these issues.

**Methods:**

An exploratory qualitative study was conducted to assess the facilitators and barriers for COVID-19 precaution dose uptake at community level across 6 different states in India. From each of the states, two districts with the highest and lowest rates of COVID-19 vaccine precaution dose uptake were selected. In each of these districts, 2 block Primary Healthcare Centres (PHCs), one with high and one with low uptake were identified. Within these block PHCs, a PHC field area with high and low precaution dose uptakes was identified. From the identified sites a minimum of four IDIs, four FGDs were conducted among the community members. KIIs of the State Immunization Officers, District Immunisation Officers, PHC Medical Officers, healthcare workers like Accredited Social Health Activist/Auxiliary Nurse Midwife were also conducted. The data was audio recorded and it was transcribed, translated and analysed using framework approach.

**Results:**

It was observed that rise in COVID-19 cases prompted the community to take the precaution dose, this along with the cost of hospitalization and the number of productive days being lost as a result of being infected resulted in vaccine uptake. The fear of non-availability of COVID-19 vaccines latter on also prompted people for vaccine uptake. While the barriers were, poor accessibility to vaccination centers, long hours of travel, poor road connectivity and lack of transportation facilities. However, the most prominent barriers observed across all study sites was that a sense of pandemic fatigue and complacency had developed both among the providers as well as the beneficiaries. Other barriers include differences in vaccination schedules and longer duration between the primary doses of some vaccines. Media was identified to be both a barrier and facilitator for Covid-19 Precaution dose uptake. Even though media played an important role in disseminating information in the beginning of the campaign, it was soon followed by the circulation of both misinformation and disinformation.

**Discussion:**

The study identified that dissemination of accurate information and community involvement at each stage of planning and implementation are crucial for the success of any campaign. Efforts should be constantly made to address and re-invent strategies that will be most suitable for the needs of the community. Therefore, in order to ensure successful vaccination campaigns, it is crucial that along with political will it is also important to have a decentralized approach with inter-sectoral coordination with different stakeholders such as healthcare workers, community members and the different departments such as the local self-governments, education department, law & order department etc. These lessons learnt from COVID-19 vaccination campaigns must not be forgotten and must be applied in future vaccination campaigns and while framing public health policies.

## Introduction

India launched one of the world’s largest COVID-19 vaccination drive on 16 January 2021 with the aim to vaccinate the adult population above 18 years of age within the shortest duration of time (1). Following the introduction of the primary doses, India further expanded the vaccination campaign in the first and second quarters of 2022 to immunize children above 6 years of age and provide an additional precautionary or third vaccine to adults who had completed primary immunization with two doses. Vaccination is being offered through a large network of COVID-19 vaccination centers (2,44,310) across the country. Implementation of a vaccination campaign of this magnitude was posed with several challenges, including community mobilization, supply-chain constraints, cold chain augmentation, training of more than 2.6 lakh vaccinators, ensuring optimum utilization of available vaccines and reaching out to hard-to-reach/marginalized populations (2). All the records were centrally captured in the COWIN electronic database, which was developed for the purpose of delivering COVID-19 vaccination. CoWIN, the “Covid Vaccine Intelligence Network,” is an Indian government web portal for COVID-19 vaccination registration, which is owned and operated by India’s Ministry of Health and Family Welfare (3). Irrespective of the challenges, the COVID-19 vaccination was executed with remarkable efficiency and speed.

As on 18 October 2022, 1,02,66,96,808 (1.02 Billion) first doses and 94,95,39,516 (949 Million) second doses of the COVID-19 vaccine were administered. However, compared to the uptake of the primary doses, the precaution dose uptake lagged behind, with only 21,75,12,721 (217 million) doses administered (4). An umbrella review conducted among healthcare workers worldwide revealed that the frequent reasons for hesitancy were sociodemographic factors such as gender, age, ethnicity, occupational factors, and vaccine-related factors such as concerns about the vaccine’s safety, efficacy, side effects, rapid development, testing, approval, and distribution. Other factors such as social pressure, collective responsibility along with distrust factors with inadequate information, and exposure to misinformation all contributed to vaccine hesitancy (5). While a qualitative study conducted in Namibia revealed that fear of death due to COVID-19, availability of COVID-19 vaccines, and influence of family and peer pressure were all identified as facilitators for the uptake of COVID-19 vaccines. The need for a vaccination certificate at workplaces and for international travel requirements were the measures proposed to increase the COVID-19 vaccine uptake (6).

Three months after the launch of the precaution dose, the government launched the “COVID-19 Vaccine Amrit Mahotsav” campaign on 15 July 2022 to boost the uptake. Under the campaign, free precaution doses were provided at all government-operated COVID vaccination centers for persons aged 18 years and above for 75 days (from 15 July to 30 September 2022) (7). Several advocacy campaigns were undertaken through electronic and print media, youth groups and NGOs, and community leaders. Irrespective of these measures, it was observed that the uptake of COVID-19 precaution dose remained very low when compared to the uptake of the first and second COVID-19 vaccination doses.

As per the WHO’s Strategic Advisory Group of Experts on Immunization (SAGE) working group, vaccine hesitancy has been defined as a delay in acceptance or refusal of vaccination despite the availability of vaccination services. Vaccine hesitancy has been found to be a complex and context-specific phenomenon that varies with time, place, and vaccine types. It is found to be influenced by factors such as complacency, convenience, and confidence (8). In the case of the COVID-19 vaccination drive in India, it was observed that the uptake of the first and the second doses of the vaccines in adults has been optimal; however, the uptake of precautionary dose remained to be poor irrespective of various efforts of the Government of India. There are many speculations regarding the factors associated with this low uptake; however, systematic scientific evidence is lacking in this context from India.

This study was undertaken with the aim to understand the facilitators and barriers to the uptake of the COVID-19 precaution dose across different states of India. The present study aimed to qualitatively explore the different stakeholders’ perspectives regarding COVID-19 precaution dose and identify vaccine hesitancy, if any, toward the precaution dose so that appropriate recommendations may be made for making policy-related decisions.

## Methodology

An exploratory qualitative study was conducted to assess the facilitators and barriers to COVID-19 precaution dose uptake at the community level. The study was conducted across six different states in India, selected from different zones to get a representative sample. The study was conducted in Uttar Pradesh (UP), Tamil Nadu (TN), Maharashtra, West Bengal (WB), Assam, and Chhattisgarh in the North, South, West, East, North-East, and Central India. The study was conducted at the selected sites by six research institutes of the Indian Council of Medical Research (ICMR) across the country and was coordinated centrally by ICMR Headquarters located in New Delhi, India. The study site investigators were ICMR scientists who were trained in qualitative research. Approval of the institutional ethics committee was obtained from all six institutes before study initiation. A multistage purposive sampling method was used to select the study sites in consultation with state immunization/district immunization officers in each state. From each of the states, two districts with the highest and lowest rates of COVID-19 vaccine precaution dose uptake were purposively selected. Based on the precaution dose coverage at the time of the initiation of the study in January 2023, the state immunization officers identified the districts as the highest and lowest precaution dose coverage districts. They then directed the investigators to select them as study sites. In each of these districts, two block primary healthcare centers (PHCs), one with a high uptake and one with a low uptake, were identified. Within these block PHCs, one PHC field area with high and one.

PHC field area with low precaution dose uptakes were identified. The details of the selected study sites are shown in Table 1.

**Table 1:** Details of study sites selected for the study.

Sl. No	State	High performing district with Precaution dose uptake and details of the PHCs selected	Low performing district with Precaution dose uptake and details of the PHCs selected
1	Uttar Pradesh^*^	Basti (54%)	Gautam Buddha Nagar (25.4%)
		High-performing PHC: Sikanderpur Low-performing PHC: Narhariya	High-performing PHC: Bisrakh Low-performing PHC: Jewar
2	Tamil Nadu	Nilgiris (33%)	Coimbatore (14.23%)
		High-performing PHC: Ketty (42%) Low-performing PHC: Ithlahar (36%)	High-performing PHC: Semmedu (38.5%) Low-performing PHC: S S Kulam (13.58%)
3	West Bengal	East Midnapore (Nandigram HD) (41.20%)	South 24 Parganas (15.63%)
		High-performing PHC: Ramnagar I (52.21%) Low-performing PHC: Nandigram II (37.21%)	High-performing PHC: Baruipur (29.69%) Low-performing PHC: Canning II (6.45%)
4	Maharashtra	Gadchiroli (30%)	Buldhana (5.3%)
		High-performing PHC: Kurud (53.1%) Low-performing PHC: Zinganoor (9.1%)	High-performing PHC: Sangrampur (8.05%) Low-performing PHC:Raigaon (0.63%)
5	Assam	Majuli district (27.53%)	Udalguri district (5.19%)
		High-performing PHC: Ratanpurmiri MPHC (51.53%) Low-performing PHC: Rangachachi MPHC (30.68%)	High-performing PHC: Udalguri (4.11%) Low-performing PHC: Khoirabari (2.72%)
6	Chhattisgarh	Kanker district (98.78%)	Baloda Bazar (28%)
		High-performing PHC: Lohattar (100%) Low-performing PHC: PV63 (40.85%)	High-performing PHC: Barnawapara (16.24%) Low-performing PHC: Moper (0.29%)

^*^In UP, the PHC level data regarding precaution dose uptake was unavailable. Hence the block PHC precaution dose data was utilized instead to identify the study sites. In High performing District, Basti the Blocks with the highest coverage was Parashurampur (3rd Dose Coverage = 65.9%) and the lowest coverage was Urban Basti (18.3%). Under these blocks there were two PHCs each. Hence from Parashurampur the best performing PHC. Sikanderpur was selected and from Urban Basti the low performing PHC Narhariya was selected based on the inputs by the District Immunization Officer. Similarly, in the Low performing District of Gautam Buddha Nagar (3rd Dose Coverage = 25.4%), PHC or Block level data was not available with the District Immunization Officer. Based on the discussions with the state immunization officer and other officials the PHCs with highest and lowest coverage were identified as PHC “Bisrakh” and PHC “Jewar,” respectively.

At these identified study areas, trained investigators first approached the community members to collect their perspectives. Investigators invited and ensured representation from all genders, differently abled/vulnerable/special groups, and formal or informal leaders (such as sarpanch, panchayat members, and school teachers) as was feasible at the study sites.

From each of the high uptake and low uptake PHC field areas, a minimum of four in-depth interviews (IDIs) were conducted (two each from those who had taken and not taken the precaution dose, respectively) to ensure equal representation from both groups. A minimum of four focus group discussions (FGDs) were conducted at both the high- and low-uptake PHC field areas (separate FGDs for male and female participants) at each state.

**Figure 1:**
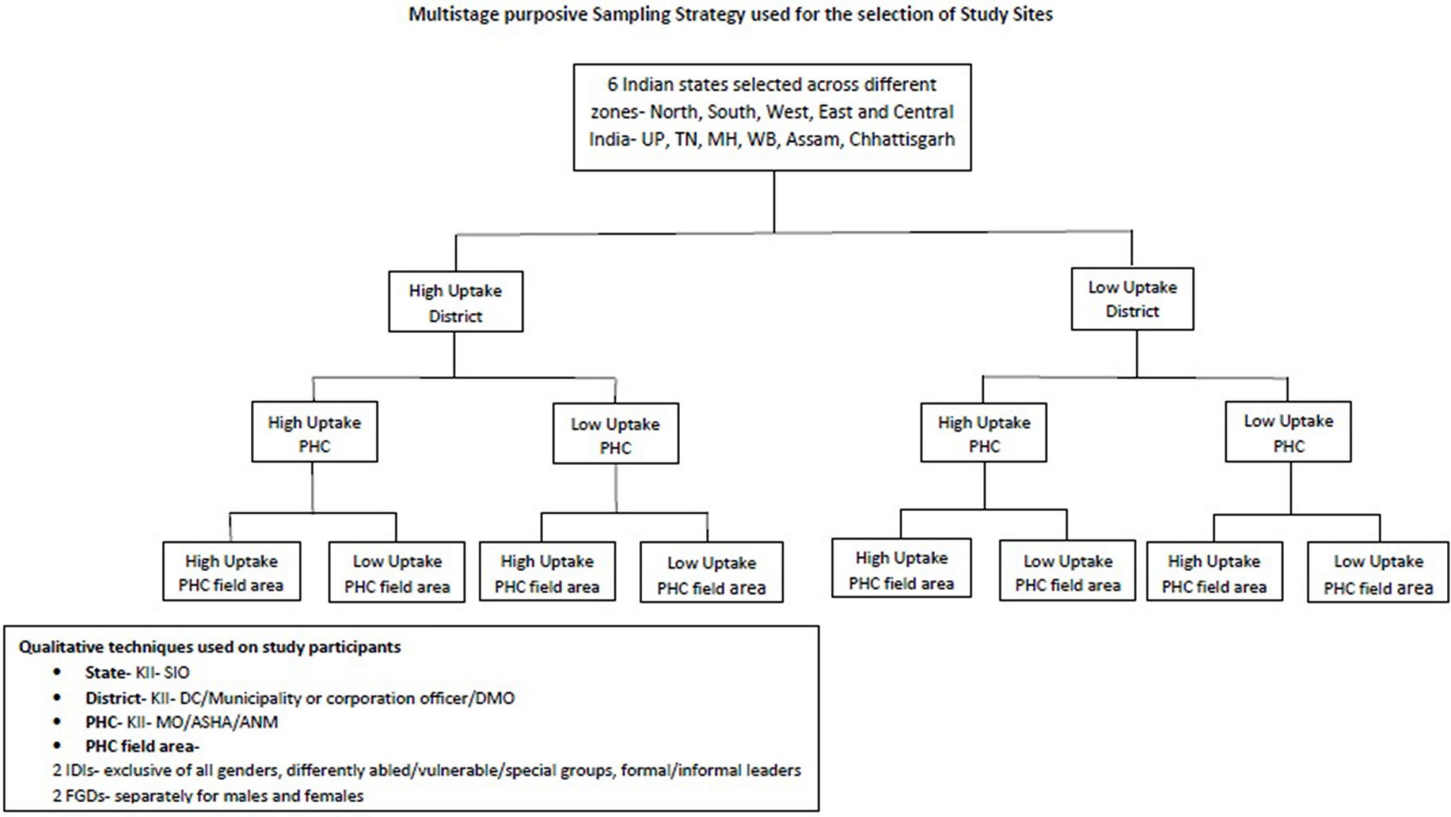
Strategy used for selection of study participants.

Key informant interviews (KIIs) of the PHC Medical Officers and healthcare workers, such as Accredited Social Health Activists/Auxiliary Nurse Midwife (ASHA/ANMs) who were actively involved in the delivery of the COVID-19 precaution dose, were also conducted. At the district level, the District Immunization Officers (DIOs) were selected for KIIs as they were primarily coordinating the activities of the COVID-19 precaution dose vaccination services. At the state level, the State Immunization Officers (SIOs) were also interviewed. Even though the data were collected from both the beneficiaries and healthcare providers, the study aimed to capture the facilitators and barriers from the perspectives of the beneficiaries only. The strategy for the selection of the participants is shown in Figure 1.

After obtaining relevant permissions, the KIIs, IDIs, and FGDs were audio recorded. In addition, field notes were also taken during the interview. The KIIs, IDIs, and FGD guides were prepared after extensive formative research. The guides were prepared based on the “Vaccine Hesitancy Determinants Matrix” developed by the SAGE Working Group on vaccine hesitancy. This comprehensive matrix was developed after reviewing various models and much discussion about factors that can influence hesitancy. A commissioned systematic review of determinants and a working group’s Immunization Managers Survey on hesitancy did not uncover any new determinants that were not already included in the matrix. The Vaccine Hesitancy Determinants Matrix displays the factors influencing the behavioral decision to accept, delay, or reject some or all vaccines under three categories: contextual, individual and group, and vaccine/vaccination-specific influences (8). Therefore, this comprehensive matrix was used to develop the IDI/FGD/KII guides to assess the factors influencing the behavioral decision to accept, delay, or reject the precaution dose of the COVID-19 vaccine.

## Analysis

At all sites, the audio files were first transcribed verbatim by the project team, which was then translated from the local language to English for uniformity. The framework approach for thematic analysis was used for data analysis. A code book was generated at each site manually while inductively going through the transcripts. Using the common code book, all transcripts in each site were coded and were then grouped together as themes under specific domains. Using the common framework provided by the coordinating site, each of the sites populated the data into the framework with summarized pieces of data in the form of quotes to enable a process of cross-comparison between different sites. The compiled data were analyzed, and conclusions were drawn.

## Results

**Figure 2:**
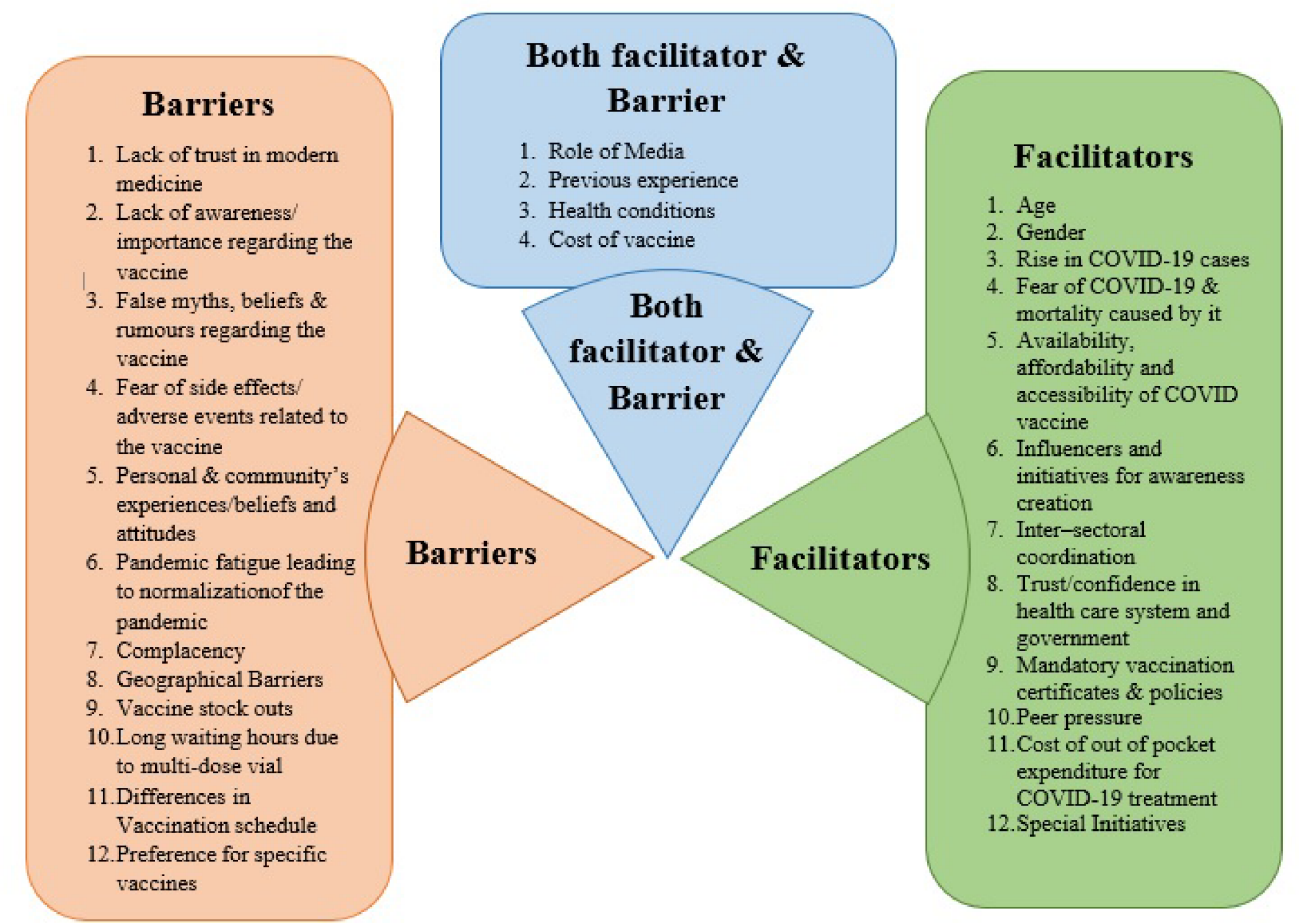
Facilitators and barriers for precaution dose uptake.

A total of 52 IDIs, 69 KIIs, and 48 FGDs were conducted across all six states. Even though a minimum of 11 KIIs, 8 IDIs, and 8 FGDs was planned to conducted at each site, they were encouraged to conduct more number of IDIs or FGDs if data saturation was not achieved. The mean age of the participants ranged from 18 years to 72 years across sites. The study design ensured equal gender distribution in both IDIs and FGDs. The participants of FGDs and IDIs consisted of a variety of people ranging from illiterate to those with primary, secondary, high school, and even graduation levels of education. It consisted of people from all spheres of life, including students, homemakers, unemployed, retired, unskilled workers such as manual laborers and farmers, and skilled workers such as carpenters and businessmen. The various themes identified under the domain facilitators and barriers for the improvement of precaution dose uptake are mentioned below, the details of which are diagrammatically represented in Figure 2.

### Facilitators for precaution dose uptake

#### Age

Age was found to be an influencing factor for vaccination uptake. In most states, older individuals were more inclined to take the vaccine due to higher risk, while in the state of Chhattisgarh, vaccination uptake was higher in the age group of 18 to 44 years and lower in the age group of 60 years and above. It was either because of their strong traditional beliefs against vaccination or because some found it challenging to reach the vaccination centers.

*“In our childhood days, we never had any vaccines and we still survived”—Male aged 74 years, IDI, non-vaccinated.*

#### Gender

Even though it is often believed that women would be lagging in vaccine uptake due to multiple reasons, such as the decision-making process and accessibility to healthcare systems, it was found that vaccine uptake among female participants was found to be better than male participants in Chhattisgarh.

*“The women in our locality reached out to me to get vaccinated. We went together as a group to the PHC and got ourselves vaccinated”—Female aged 46 years, FGD, vaccinated.*

Male daily wage earners from low-performing districts stated that vaccination was a waste of time and waiting at centers resulted in the loss of wages. They considered vaccination as non-urgent and showed a sense of reluctance and procrastination to be vaccinated.

#### Rise in COVID-19 cases

The state health officials were of the opinion that the increase in COVID-19 cases across different states in India as well as globally also prompted the community to take the vaccine.

*“Recently there were talks about the rise in numbers in China. During that time there was surge in vaccine demand and whatever vaccines were available got exhausted so rapidly that we did not have to do anything”—State Immunization Officer, KII, vaccinated.*

### Fear of COVID-19 and mortality caused due to it

*“I was admitted with COVID-19 for 11 days alone and took more than 40 injections. I was about to die. So, not to get COVID-19 again, I have taken all 3 doses of the vaccine”* — *Female, FGD participant, vaccinated.*

Fear of the COVID-19 disease and deaths caused by it during the first and second waves of the pandemic still persisted in the minds of the people at selected sites in Tamil Nadu, Maharashtra, Assam, Uttar Pradesh, and West Bengal. Some people felt that, by taking the third dose, they were completing the full course of vaccination and would be completely protected. They hoped that they would not have any recurrences and that, even if they got infected, the severity would be limited. Additionally, some of the participants feared the financial burden that they would have to bear if they got infected with COVID-19 since the treatment was costly and they would have to lose their daily wages because of it.

### Availability, affordability, and accessibility of COVID vaccine

At some of the sites (Maharashtra and Chhattisgarh), special efforts were taken by the medical officers and their teams to deliver the vaccine at a time and place that was most convenient for the people. Outreach vaccination camps were conducted at construction sites, paddy fields, etc.

*“We arranged vaccination either early morning or late evening, to encourage more people to get vaccinated without losing their daily wages”*—*Medical Officer belonging to a high performing district & PHC, KII.*

In Chhattisgarh, due to its hard terrain, people were hesitant to travel to the health center. Many of those who took the primary doses were reluctant to travel again for the precaution dose due to the distance. Hence, the village administrative agency (panchayat) organized free transportation services for the beneficiaries to commute for vaccinations.

*“The Sarpanch arranged vehicles for them to reach the vaccination center such as pickups or tractors. Because of this, huge number of people would come in vehicles”*—*Medical Officer, belonging to high performing district & PHC, KII.*

The provision of free vaccines in the government health system while payment is a requirement at the private hospitals motivated them to get vaccinated. An IDI participant from a low-performing district who is vaccinated stated the following: *“If money had to be paid, then not everybody would take the vaccine. We are poor people. How could we be able to get vaccine by paying for it?”*

Additionally, the cost of vaccination was reportedly used by health officials as a motivating element to persuade people to get vaccinated in Chhattisgarh. They claimed that, when people began to show reluctance, the administrative department announced that the vaccine would be charged after a specified time frame. As a result, it compelled a lot of individuals to get vaccinated.

*“When the third dose was ready for use, the collector issued a letter that, after a certain number of days, the vaccines will be available upon payment. The rates were decided that one dose will cost Rs 800 or Rs 1,000 for Covaxin and Covishield. This had a huge impact on people. They understood that, they will not get the free vaccine latter, then why not take the third dose before that?”—Medical Officer, belonging to a high performing district & PHC, KII.*

#### Influencers and initiatives for awareness creation

The study identified a series of influencers ranging from ASHAs, ANMs, medical doctors, family, friends, neighbors, local non-governmental organizations, and women self-help groups to district collectors, politicians, and film stars. In Maharashtra, Assam, UP, Chhattisgarh, and TN, the ASHAs and ANMs went house-to-house to motivate people. This provided an excellent opportunity for the community members to discuss with the healthcare workers about the vaccine and their misconceptions, fears, anxiety, and doubts that they had regarding the vaccine. This personal attention created a lot of confidence among them, resulting in vaccine uptake: *“ASHAs and ANMs played an important role in creating awareness among public by their household visits” District Health Officer, high performing district.*

In addition to providing vaccines and identifying and tracking the target group, the ASHAs mobilized the community for vaccination sessions. At different sites, they were assisted by other influencers such as doctors, school teachers, headmasters, village heads, and religious leaders. They were all respected and trusted by the community. The religious places and schools contributed as sites for vaccination and dissemination centers, respectively. One respondent stated the following: *“When we visited the temple they used to announce to get ourselves vaccinated. This motivated us to get vaccinated”—Male participant from a high performing district who has taken the precaution dose, IDI.*

In Tamil Nadu, villagers were motivated to take the vaccine when they saw famous personalities such as film stars and local, regional, and national leaders take the vaccine. *“After watching the actors and Prime minister getting vaccinated, we got it too”—Male participant of an FGD from a low performing district who has taken the precaution dose.*

The district collector (DC) too was identified as an influencer who created awareness and also motivated the different departments, such as education, police, agriculture, health, and others, to take the vaccine. The DC ensured that employees in different departments were vaccinated and they would disseminate information to others.

#### Inter-sectoral coordination

Inter-sectoral coordination among the public health system, local administrative bodies, media, and local stakeholders played a very important role in improving vaccination coverage, especially in the state of Maharashtra. The Panchayat Raj system not only facilitated the implementation of the vaccination program but also increased its outreach through community mobilization.

*“Support of administrative bodies, panchayat raj in reaching the grass root level and helping the health system to conduct the vaccination camps was very important” shared the District Health Officer of a high performing district in Maharashtra.*

In sites with high coverage, it was observed that extensive pre-planning and micro-planning were conducted with the involvement of different sectors. Moreover, the “political will” supporting COVID-19 vaccination played a crucial role.

### Trust/confidence in the healthcare system and government

One of the major factors that favored the uptake of COVID-19 vaccine was the trust that people have in the healthcare system. This trust was achieved not by mere force but rather by the dedication of the healthcare workers. In a country where most of the population lacks adequate health literacy, healthcare workers play an imperative role in guiding the health-seeking behavior of common people. Healthcare workers were actively involved in driving out people’s fear regarding vaccination misconceptions and advised them not to worry whenever they experienced minor side effects post-vaccination. The healthcare workers accepting accountability for adverse events following vaccination was a key element in encouraging people to get the dose. A participant stated the following:

*“In AIIMS hospital, in order to convince the people to get vaccinated, the doctor even assured that if anything happens, they will take the whole responsibility of their family”—Female participant from a high performing district who has taken the precaution dose.*

The community’s trust in the government vaccination system also played an important role in the vaccination campaign. Female participants of an FGD in Maharashtra stated that they trusted not only the health system but also the local government so much that they were even willing to take up a fourth dose of the COVID-19 vaccine if it comes in future.

#### Mandatory vaccination certificates and policies

Mandatory vaccination certificates for travel and availing government schemes were noted by the respondents as one of the factors for improving vaccination coverage. One key informant from Maharashtra who took the precaution dose stated that the following:

*“Incidence of COVID-19 was high and people were denied ration (food supplied by public distribution centers) and to travel by bus without vaccination certificates. So, people were getting vaccinated in order to avail ration, to get the bus pass because vaccination certificate was made compulsory.”*

The community members claim that the “no ration” policy and the inability to travel to other districts without vaccination certificates instilled fear in them and forced them to get vaccinated even against their will at times. One of the participants remarked the following:

*“We had no other option. It was told that if we do not take it, we will not get ration. It was coercion, if we did not comply.” Male participant from Chhattisgarh a low performing district who has taken the precaution dose.*

Another participant stated that they were “forced” to get vaccinated since it was the company’s policy that all must be vaccinated:

*“The company is demanding that the third vaccine should be taken, only then I can pass through the gate. So, I have taken it”—Male FGD not taken a precautionary dose.*

Since the precaution dose was not mandatory, it was observed that most people were not willing to take it. Hence, the question of whether it needs to be made mandatory was raised, and there were mixed responses. Some felt that it was a personal choice based on individual assessment of the risks and benefits associated with it, while others felt that it should be mandatory because it is in the best interest of the community as a whole. However, according to the state immunization officer, it was important to give the right information to the community and help them make the decision for themselves:

*“We should tell people about the need for vaccination and if not taken what will be the ill effects of that. If we are able to give complete information, then people will definitely take the vaccine. Even if they do not take the vaccine, then it is their personal choice. It is necessary that we give full information for them to make the right decision.”*

#### Peer pressure

Peer pressure played an important role in vaccine uptake. It was found to work well among the youths in colleges and the villagers. When they saw their neighbors take the vaccine and after listening to their experiences, some of the villagers got motivated to take the vaccine and also complete the vaccination schedule. As stated by a female participant who had taken the precaution dose during an FGD, *“There [sic] was competition in the society to take vaccines. We used to think that all members living next door have taken the vaccine, we still have 2 members unvaccinated in our family. We also should complete vaccination of our family immediately.”*

#### Cost of out-of-pocket expenditure for COVID-19 treatment

On the flip side, when health providers explained the risk of infection by not taking the vaccine and the associated treatment costs, some beneficiaries decided that the benefit of avoiding out-of-pocket health expenses outweighed the benefit of not taking the vaccine.

*“People thought that if they got infected, they will have to get admitted, loose their daily wages and even spend money for their treatment. So, people decided to take the vaccine”—Female participant from a high performing district who had taken precautionary dose, IDI.*

#### Special initiatives

It was observed that, since health is a state subject, in states such as Chhattisgarh, Assam, and Tamil Nadu, special initiatives were carried out to increase vaccine uptake.

In the high uptake district of Majuli in Assam, the Chief Minister implemented the *“Har Ghar Dastak,”* a mass campaign for increasing vaccine coverage through house-to-house visits.

*“The Cabinet Ministers along with senior officials visited their respective districts for supervising the district administration for ensuring the successful implementation of “Har Ghar Dastak”—Medical officer.* However, in the Udalguri district, this approach was unable to reach out to all the beneficiaries.

In Assam, mobile units for vaccination were deployed to ensure that no one was left out. Moreover, the vaccination programs were planned in a strategic manner by inviting eligible individuals in groups to avoid long queues. In addition, vaccination was linked with other government schemes and services, such as the food security scheme, where all family members had to be vaccinated in order to claim their food rations. Meanwhile, in Majuli District, a mandate was made for shopkeepers and customers that every shopkeeper must be vaccinated to open their shops, and the customers need to be vaccinated in order to purchase from the shops. This mandate created fear and prompted people to get vaccinated.

In Tamil Nadu, the district authorities appreciated the primary health centers that achieved 100% coverage with the second dose. This motivated them to maintain the same momentum for the precaution dose as well.

*“In the district, our block won the award for achieving 100% vaccination, and the collector presented us with a shield and trophy. We felt that our efforts were not a waste, and in our block death rates were reduced compared to others”—Medical officer of a low performing PHC, KII.*

In a high-performing district of Chhattisgarh, various initiatives were taken to remind people of their precaution dose dates.

*“We used to maintain a register. If a person walked in to enquire, his phone number would be recorded and we would contact him whenever he was due. Also, a reminder slip was given at the time of their primary doses and we asked them to keep it till their next dose”—District Immunization Officer of a high performing district, KII.*

The other initiatives conducted were organizing awareness camps at night, reminder calls for vaccination, writing slogans on walls, conducting rallies, measuring vital parameters such as blood pressure and blood sugar level before administering vaccines, and sharing positive experiences with community members.

### Both facilitator and barrier

#### Role of media

It was observed that the community was influenced by different types of media, such as newspapers, mobile apps, TV, and public information systems, but the major source of influence was identified to be social media. In all the sites, the response toward the impact of media on vaccine uptake was mixed. On the one hand, Social media was used as a source to disseminate information regarding the vaccine and its availability and types of vaccine at the different vaccination sessions. However, on the other hand, it aided in spreading rumors, myths, and fear in the community. Hence, it was identified to be both a facilitator and a barrier.

Apart from these effects, the media has also helped in building trust among the public regarding the safety and effectiveness of the vaccines.

“*Watching the news on TV that actors, ministers, PM, and CM were getting vaccinated convinced us that the vaccine is safe”—Male FGD participant who had taken the precaution dose from a high performing district.*

Television and WhatsApp were identified as important tools for the dissemination of information due to wide outreach.

*“Every house has a TV. Television awareness reaches even those houses where health workers fail to reach”—Female of an FGD from a low performing district who has taken the precaution dose.*

*“In a WhatsApp message, VHN sister told me about the vaccine availability and vaccine camp location”—38-year-old IDI female participant who has taken the precaution dose from a low performing district.*

Social media was also used as a tool for community mobilization. People of all ages posted the vaccination picture on their social media posts, and many others were actually motivated by it.

*“As soon as we got vaccinated, we posted the picture on Facebook and WhatsApp as a status update. Seeing this many of our friends got vaccinated”—Male who has taken precaution dose belonging to high performing district, FGD.*

It was observed that, initially, there were no challenges, but, as time progressed, rumors or unfavorable information regarding the vaccine’s side effects began to circulate and gain attention on social media, notably WhatsApp.

*“In beginning there was nothing, no hesitation. It was something in middle,* i.e.*, in between one and a half to two months later, there were antisocial elements, through WhatsApp that entered percolated into our district as well as other districts and then, problems started.” District Immunization officer of a high performing district, KII.*

Some media outlets sensationalized adverse reactions, leading to confusion and mistrust among the public. The common rumors were that the vaccine resulted in decreased immunity and long-term side effects such as heart attack, paralysis, kidney problems, early aging, infertility, and impotency after vaccination. Some even claimed that there was no disease called COVID-19, and the pandemic was all fabricated.

*“There were things on WhatsApp… like you will not be able to have children after taking the vaccine or you might even die”—Female participant of an IDI from a high performing district who has not taken precautionary dose.*

On the converse, the media’s emphasis on the rapid spread of the COVID-19 infection and the lack of hospital beds also fueled people’s fears of contracting the disease and dying, which persuaded them to get the vaccination as a precautionary measure. The state and local health departments took a variety of initiatives to dispel the rumors, including immunizing health workers first in front of community members to set an example, forming a social media group with village leaders and youths to discuss and debunk myths, making public announcements through street plays, and distributing pamphlets, microphone announcement, and publicizing vaccination schedules to the community via social media and newspapers.

*“We immunized some of the health care staff in front of common public and demonstrated that nothing happened. Also, we made several What’s app groups with elders, sarpanch and youths. If they had any misconception, we clarified”—Medical officer of a high performing PHC in a high performing district, KII.*

However, participants who had not received the precautionary dose noted the absence of aggressive advertising during the third COVID-19 vaccination dose via the media or other forms of communication. The only means to be notified of the third vaccination dose was via a notification on mobile devices and through lay health workers.

*“The third dose was not taken seriously. There was no media advertising for it”—42-year-old male participants from low performing district who had not taken the precaution dose, IDI.*

*“No sir, there is no coverage on any of the news channels. The PHC officials kept me updated time to time. The media did not advertise that much about it”—27 Year-old male who had taken the precaution dose from a low performing district.*

### Previous experience

Previous experiences with vaccination, whether good or bad, influenced the decision-making process and hence acted as both a facilitator and a barrier. The positive experience of the previous doses of vaccination by self, family, or significant others is a powerful facilitator for the uptake of precaution doses.

Many of the non-vaccinated interview participants expressed their concern that bad experiences among their friends and relatives in terms of side effects such as pain and fever, followed by loss of productivity for 2 to 3 days, were the reasons for them to not take the vaccine. Moreover, in the present scenario, where there is minimal or no COVID transmission, they are not ready to undergo the process of vaccination again. Hence, since the perceived risks overweighed the perceived benefits, they avoided taking the precaution dose.

*“I feel that I am not active like before, I took the COVID vaccine. As there are no COVID cases, I do not want to take the risk again”—Male participant who has not taken precaution dose, IDI.*

#### Health conditions

It was observed that having co-morbidities was found to be both a facilitator and a barrier. Having a co-morbidity put them at a higher risk and motivated them to get vaccinated, while, on the other hand, it created hesitance among others. Having surgery or being pregnant were found to be factors that delayed the uptake of the precaution dose.

*“I took two doses after which I underwent operation, which is why I could not get third dose. I was informed to take but I got operated so did not take”—55-year old female.*

#### Cost of vaccine

In a country like India, where a majority of the population lives on limited resources, the affordability of vaccines is a great challenge. Hence, vaccines being provided by the public healthcare system free of cost is a great boost for vaccine uptake.

*“If the vaccine was chargeable, we could not afford the cost of vaccine. Since, the government made the vaccine free for public we took it”—Female IDI participant from a high performing district who has taken precaution dose.*

However, the free supply of vaccines was also found to have a negative side since it created suspicion among the people regarding its quality. They felt that the government’s free supply of vaccines may not be of good quality, and those provided by private facilities were more effective. Health workers were of the opinion that people might disregard the efforts of the public healthcare system and that the whole vaccination drive could end in a disaster. They felt that it was their duty to educate the public and make them understand that there is no difference in the vaccines provided by the public and private facilities. However, irrespective of these barriers, the majority of people interviewed had trust in the government’s healthcare system and the vaccination policy. Irrespective of their culture, religion, and community, they all believed in the immunization program of the government. Even though, in general, people never questioned openly about the quality of vaccines, they did have their doubts in this regard. However, the free availability of vaccines was considered to be more as a facilitator rather than as a barrier.

*“Some people in our area told that vaccine given in the government facilities could be less effective since it is given free of cost. But I do not believe it. Whatever government provides will only be good”—Male IDI participant who took the precaution dose.*

### Barriers to precaution dose uptake

#### Lack of trust in modern medicine

People’s belief in modern medicine played a big role in vaccine acceptance. Certain sections of the population lacked faith in modern medicine, and hence, they were hesitant to use all vaccines, including COVID-19. One such community was the tribal community of Semmedu in Tamil Nadu, which was not even willing to take up the primary doses of the COVID-19 vaccine. This community was not interested in vaccination from the start, and it took a collaborative effort by the panchayat, revenue, police, and health department to convince them to take the primary dose of COVID-19. As one ASHA worker stated following:

*“Some even climbed up the tree to avoid vaccination. Later we convinced them. Village Administrative Officer, police, block medical officer and we all went to talk to them. We even told them they would not get their ration if they did not get the vaccine”—ASHA worker, vaccinated and belonging to a low performing district, KII.*

Similarly, hesitancy was also observed toward the precaution dose. Hesitancy when coupled with the complacency that developed at all levels in the health system during the time of precaution dose introduction, it was observed that the system did not make significant efforts to ensure the precaution dose uptake by the tribal community.

#### Lack of awareness/importance regarding the vaccine

Lack of awareness, especially regarding the importance of vaccines and its impact on the community’s wellbeing, was identified as a barrier. As stated by a vaccinated respondent, “*some of the community members are not aware about the importance of vaccine and how it works,”* the concept that vaccines do not prevent the disease but only prevents severe infection and death is unknown to most people. Therefore, there is a need to create awareness about the vaccine, how it works, the dosing schedule, where it is available, and how it would benefit the community. In the FGD conducted in the low uptake district of Assam, most of the members responded that they did not take the precautionary dose as they were not informed and were not aware that the vaccines were being provided in the village. This was the situation in UP as well. As some of the participants stated that the following: *“Many people do not know that there is a booster dose”—34 year old Male.*

#### False myths, beliefs, and rumors regarding the vaccine

The spread of false rumors and myths in the villages by word of mouth created apprehension, fear, and mistrust in the minds of the people. The most common rumors circulating was that if one got vaccinated, it would lead to infertility, impotency, overall reduction in immunity, increased occurrence of morbidities, and long-term side effects such as myocardial infarction, stroke, renal failure, early aging, and mental disorders. The circulation of rumors and myths was found to be more among the minority population and also among sects of the community that were religiously or politically afflicted, especially in the state of UP. Moreover, among men, there was a hesitation to get vaccinated as there was a rumor that, if one took the vaccine, they would not be able to consume alcohol.

### Fear of side effects/adverse events related to the vaccine

People who had any side effects with the first two doses were largely hesitant to take the precautionary dose. Most of the women interviewed complained about body pain and back ache from the time of getting vaccinated till date. This has impacted their day-to-day chores and child care.

News of sudden death among famous personalities following vaccination also created fear and resistance. In Tamil Nadu, the death of the famous actor Vivek resulted in fear in the minds of the people.

*“Actor Vivek’s death was caused by COVID vaccine and this terrified us”—Female FGD participant from a high performing district who has not taken a precautionary dose.*

#### Personal and community’s experiences/beliefs and attitudes

The role of personal beliefs, irrational fear, and community’s beliefs also played an important role in vaccine uptake. The health officials from Chhattisgarh described an incident in which communities barred them from entering villages and prevented them from carrying out the vaccination drive:

*“There is a village nearby, there they completely denied entry. Some function was going on in the village. They said you can check BP, sugar as well as malaria but we are not ready for this [COVID vaccine]”—District Immunization Officer of a high performing district, KII.*

#### Pandemic fatigue leading to the normalization of the pandemic

At all sites, it was observed that people expressed a sense of pandemic fatigue. They were all trying to normalize it by stating that COVID-19 is no more a pandemic. This perception of the villagers hindered vaccine uptake, and a medical officer of a low-performing PHC stated the following:

*“People think that there is no more COVID-19. So, why to get 4–5 days fever after taking precaution dose unnecessarily. Even the focus on vaccination drive is diluted.”*

A similar point was also mentioned by a male participant in an FGD who belonged to a low-performing district who had not taken the precaution dose stated the following:

*“I have taken both 1st dose and 2nd dose…but… I do not feel the need for the 3rd dose ….even channel news, newspapers mentioned that the spread of Corona has reduced now…so thought that two doses will be enough…and so ignored the booster dose.”*

The news about relaxing the restrictions imposed due to the COVID pandemic led to an enhanced false sense of security against COVID among villagers. The focus of the public health system was diverted to their routine activities while reducing the number of COVID-19 cases.

#### Complacency

One of the reasons for the community being complacent was that the disease caseload had decreased, and so had the severity. In addition to decreased caseload, people were getting re-infected even after taking the vaccine, and since it was not compulsory, people did not feel the need to get vaccinated. Moreover, some of the participants felt that they were already being protected with the two doses. They felt that the precautionary dose was not provided like the first and second doses of the COVID-19 vaccine along with the pro-active participation of different departments. Hence, they felt a precaution dose was not a necessity.

It was observed that, over the course of the precaution dose, the complacency of family members and significant others affected the uptake of the vaccination by others. It was also observed that, if the influential local leaders felt that the vaccine was not necessary, it reflected in all the arrangements/efforts to conduct a vaccine campaign. Hence, complacency was felt not only among the beneficiaries but also among the local leaders and other stakeholders as well. As complacency gradually emerged in the community in view of the reduction in the cases and deaths, the general uptake level of precaution dose decreased.

*“Even, he (pointing to the panchayat leader) is not serious this time in arranging vaccine camps. But, he did a fantastic job during second COVID wave”—Female participant who had not taken the precaution dose from a low performing district, IDI.*

### Geographical barriers

Respondents stated that there are some difficult-to-reach areas where road networks are narrow and only two-wheeler vehicles can travel, while other areas are located across the river, making it difficult for people to reach the vaccination site. Furthermore, during the rainy season, the road’s condition deteriorates, preventing many people from visiting the vaccination point. On the other hand, in Chhattisgarh, there are Naxalite areas where transportation is inadequate and health workers are afraid to go.

*“Some areas in the interior were hard to reach and we had to cross the river and then there were some places where one had to go either on bike or on feet. Also, there were lack of transport facilities in the Naxalite area and people were afraid to go there”—Medical officer of a low performing district, KII.*

#### Vaccine stock-outs

At certain sites (Chhattisgarh, UP), participants complained that precaution dose vaccines were not available at all at the PHCs or, at times, the number of beneficiaries was more than the available vaccine doses. Stock-out positions led to people refraining from getting vaccinated.

#### Long waiting hours due to multi-dose vial

Another barrier mentioned by beneficiaries was the long wait time at the healthcare facilities. Since a single vial contains 10 or 20 doses, beneficiaries were asked to come in groups of 10 or to wait for 10 people to arrive. Beneficiaries frequently reported returning without being vaccinated. As a result, many of them were hesitant to come back to the center.

*“it is not like that vaccination will get completed in five minutes. We have to come in a group of ten-fifteen people, then it takes two to three hours… so sometimes if 10 people are not available there, then we have to return unvaccinated”—Male FGD participant from a low performing district who had not taken a precautionary dose.*

#### Differences in vaccination schedule

According to health officials from Chhattisgarh, differences in vaccination schedules based on the vaccine brand influenced precautionary dose uptake. In the case of Covaxin, there was a 28-day interval between the first and second doses, while for Covishield, the interval between doses was 12–16 weeks. Since the third dose was given 9 months after the second dose, those who had taken Covaxin completed their schedules faster than those who had taken Covishield. As a result, many people demanded Covaxin to finish their schedule faster. Delaying or missing the second dose also affected the precaution dose uptake.

*“I could not understand but most of the people wanted Covaxin. When I tried to enquire I found that because the two doses of Covaxin was only 28 days apart people preferred it thinking their schedule will complete soon and they will be protected. While for Covishield there was a long gap between two doses and they would have to wait long for taking the precaution dose in order to complete the schedule”—Medical officer of a high performing district, KII.*

#### Preference for specific vaccines

Even though no specific concerns about pharmaceutical companies were voiced by participants, they, however, expressed concerns about the efficacy of Covaxin and Covishield.

*“In the PHC most of the people got covishield, covaxin came later. As people used to say that covaxin is not that much effective, I found covishield more effective”—Male IDI participant from a low performing district who had taken the precaution dose*.

Many of the beneficiaries, according to health officials, requested Covaxin because there were minimal or no reported side effects as opposed to Covishield, which had the most common side effect of fever.

*“Sir, they used to ask for Covaxin because they did not get fever after that. But for Covishield, they used to have fever for 4–5 days. So they wanted to get vaccinated with Covaxin”—ANM of a high-performing district, KII.*

## Discussion

The study was able to identify various facilitators and barriers for precaution dose uptake by the community across different states of India. It was observed that some factors played the role of both a facilitator and a barrier. Understanding these factors is crucial for decision-making so that appropriate targeted interventions/initiatives can be launched to overcome these barriers and increase vaccine uptake.

It was observed that the increase in COVID-19 cases prompted the community to take the precaution dose; the COVID-19 case increases along with the cost of hospitalization and the number of productive days being lost as a result of being infected resulted in vaccine uptake. Mertens G et al. have shown that fear of COVID-19 was a significant predictor for willingness for vaccination against COVID-19, even when measured after controlling for anxious personality traits, infection control perceptions, risks for loved ones, self-rated health, media use, and demographic variables (9). The fear of COVID-19 coupled with fear of non-availability of COVID-19 vaccines later on also prompted people for vaccine uptake.

A wide range of influencers spanning from ASHA workers and local leaders such as village heads and panchayat members to district collectors were identified. It was observed that community ownership was crucial for the success of COVID-19 vaccination campaigns. This has been true for routine immunization as well. Literature has shown that, to improve vaccine acceptance among communities, a bottom-up approach to planning and program implementation by community involvement is crucial for the success of any vaccination program (10).

Accessibility to vaccination centers in the rural and tribal areas due to long hours of travel, poor road connectivity, and lack of transportation facilities were all identified as barriers. This finding was similar to a district-level analysis of COVID-19 vaccine coverage, where it was observed that districts where higher concentrations of marginalized communities lived had much lower vaccination rates (11). However, special initiatives were taken to ensure equitable access by the local panchayat and healthcare system, such as creation of mobile units, conducting camps in the evenings or in places nearest to where people lived. Irrespective of these efforts taken, it was observed that it lacked uniformity.

It was observed that more than the vaccine’s safety and efficacy, the trust in the healthcare system and the government’s political commitment were identified as facilitators for vaccine uptake. However, in other parts of the world, such as the USA, the strongest factors associated with and indicative of vaccine willingness were the COVID-19 vaccine’s safety and efficacy and the belief that, by taking the vaccine, they were protecting themselves and others (12).

One of the most prominent barriers observed across all study sites was that a sense of pandemic fatigue and complacency had developed both among the providers as well as the beneficiaries. Pandemic fatigue is defined as the demotivation to follow recommended protective behaviors that gradually emerge over time, and it is often affected by a number of emotions, experiences, and perceptions. It is manifested by an increasing number of people not sufficiently following recommendations and restrictions, decreasing their efforts to keep themselves informed about the pandemic and having lower risk perceptions related to COVID-19. Fatigue and complacency occurred as a result of the longevity of the pandemic (13). With the decrease in the number of cases, the perceived threat decreased in the minds of the people, and they started to “normalize” the pandemic. Perceived threat, coupled with personal, social, and economic losses suffered due to pandemic lockdowns or restrictions, further resulted in complacency. It was not only experienced by the community but also by the healthcare providers and administrators. Perceived threat was manifested by the re-direction of funds and control activities toward other priority diseases other than COVID-19.

The community wanted to move ahead with life, and they felt that they were sufficiently protected with two doses of the vaccine and that the third dose was unnecessary. With the emergence and evolution of the omicron sublineages, the severity of the diseases in terms of morbidity and mortality also decreased, which also resulted in the reduction of fear, further leading to complacency. Complacency coupled with the news of strokes, heart attacks, and sudden death among famous personalities following vaccination, along with the circulation of false myths and rumors in the media, all prevented precaution dose uptake. The COVID-19 “infodemic” complicated the process of searching for and accessing reliable information due to its overabundance, of which some are true, some false, or even misleading. It has been shown that misinformation and disinformation have resulted in the reduction of vaccine acceptance in the community (14).

Personal past experiences with the primary doses and their interaction with the health system shaped their decision-making process. They were also influenced by the community’s experience with COVID-19 vaccination. The occurrence of immunization stress-related responses (ISRRs) consisting of a range of symptoms and signs arising around immunization that are related to “anxiety” and not due to the vaccine product or due to its quality or error in the immunization program was also identified as a barrier (15). Therefore, there is a need to implement prevention strategies which should include proactive communication, management of social media, and creating in-clinic environmental strategies. These strategies should include active screening to identify those with an increased risk of ISRR, high levels of needle fear, or history of vasovagal reaction. Age-appropriate pain management strategies should be made available for all recipients. Following screening, targeted interventions should be provided for those experiencing ISRR, such as muscle tension for vasovagal reactions, reducting vaccine recipients’ fear, increasing comfort, and avoiding the contagion of fear and misinformation (16).

Differences in vaccination schedules and longer duration between the primary doses of some vaccines resulted in the delay of the uptake of precaution doses. Moreover, preferences for specific vaccines were also identified as barriers at selected sites. Initially, only homologous booster vaccines were permitted, and this too acted as a barrier to precaution dose uptake. Timely approval of heterologous precaution dose introduction would have facilitated much higher uptakes for precaution dose (17). Moreover, in the recent meeting held in March 2023, WHO’s Strategic Advisory Group of Experts on Immunization (SAGE) stated that, since most people have been vaccinated or immunized or both, the precaution dose may be reserved for people at high risk only. Hence, countries should consider their specific context while deciding whether to continue vaccinating their low-risk groups without compromising on the routine immunization status (18). Studies have shown that the side effects following vaccination by specific vaccines resulted in people either differing or delaying the next dose (19). Even though the CoWIN platform provided an opportunity to register, select, and choose their preferred vaccination center, some people still preferred to wait till their vaccine of choice was made available at the vaccination sites closest to their homes, resulting in a delay in uptake (20).

Evidence has shown that vaccine uptake increases when cost is removed as a barrier (21). Therefore, to ensure that cost does not act as a barrier, the Government of India provided vaccines through its public health systems freely. Through the 75-day long “COVID Vaccination Amrit Mahotsava,” over 15.92 crore precaution doses were administered. Even though this campaign led to an improved coverage from 8 to 27% (22) the uptake still remained low, showing that cost alone is not the single most factor affecting decision-making. A nation-wide cross-sectional study was carried out across different states in India to identify the sociodemographic determinants of willingness and extent to pay for the COVID-19 vaccine. The study revealed that the majority of the participants stated that they were willing to pay only up to 50% of the cost of the COVID-19 vaccine, and income was observed as a precursor predictor for their willingness to pay. It was also observed that being single, belonging to the higher-income group, and having a less family size were found to be having significantly higher odds of willingness to pay for the COVID-19 vaccine (23). Other barriers are as follows: poor access to vaccination sites in the form of lack of transportation, bad roads worsened with the rainy season, and lack of caretakers to accompany the older persons to the health facilities, especially those living in the rural/tribal areas or in the outskirts. Studies have shown that there is a link between population density and vaccination site accessibility. Neighbourhoods in ‘sparse’ or ‘dispersed’ settings are typically found to experience poorer accessibility in the form of greater average journey times, poor connectivity resulting in inequalities. (24) This coupled with long waiting hours at the vaccination sites and sometimes even having to return couple of extra times to pool minimum number of beneficiaries in order to avoid vaccine wastage was also identified as a barrier (25). However, it was observed that this rule was not being followed at most places, resulting in long waiting periods and people becoming hesitant to vaccination.

Media was identified to be both a barrier and a facilitator for COVID-19 precaution dose uptake. Even though the media played an important role in disseminating information in the beginning of the campaign, it was soon followed by the circulation of both misinformation shared by people who did not intend to mislead others and those who shared disinformation, which was deliberately created and disseminated with malicious intent. This was spread through both social media and other channels, and it affected people’s confidence in the COVID-19 vaccine. It was observed that misinformation often arises when there are information gaps, and it is human nature to seek reason and fill in these gaps. Both misinformation and disinformation circulated focussed on vaccine development, safety, effectiveness, and COVID-19 denials (26), which was found to affect vaccine confidence, resulting in low vaccination uptake rates. Hence, there is an urgent need to address these issues by monitoring the different media, listening, analyzing the reasons why misinformation is circulating in the community, and planning appropriate messaging strategies.

A major limitation of the study was that, it only recorded the facilitators and barriers for precaution dose from the client’s perspective, and the barriers and shortcomings from the providers’ side were not captured, which was because this study was carried out to get a purview into the community’s perceptions and needs for increasing their uptake of the precaution dose, so the policymakers could plan necessary interventions to increase the same. The strengths of the study include the fact that it was conducted across the six different zones of India and that it captured the perspectives of people from all walks of life. The study identified that dissemination of accurate information and community involvement at each stage of planning and implementation is crucial for the success of any campaign. Policymakers and program managers, while implementing such mass vaccination campaigns, should constantly be aware of the community’s needs, gaps in vaccine delivery, and information voids that result in vaccine hesitancy. Efforts should be constantly made to address and re-invent strategies that will be most suitable for the needs of the community. Even though special initiatives were launched in certain states to reach the unreached, it was observed that these initiatives were not uniform.

Therefore, to ensure equitable access to vaccines in the future, a detailed micro-planning exercise with a special focus on mapping, tracking, and follow-up of vulnerable populations, such as migrant population and slum dwellers, needs to be carried out. Innovative initiatives such as mobile vaccination clinics need to be sent to hard-to-reach areas to ensure that no one is left behind. Incentives may be provided for outreach workers who carry out community mobilization and also for healthcare providers who are working in hard-to-reach areas. The behavior change communication campaigns are as important as vaccination campaigns. Hence, every opportunity is used to create awareness regarding the benefits of vaccination using innovative techniques and the appropriate use of social media. It is also crucial that the message that the COVID-19 vaccine prevents mortality and severe morbidity and does not prevent re-infection must be clearly conveyed to the community. Along with media, the role of healthcare workers and the community were found to be crucial for increasing vaccine confidence. Therefore, to ensure successful vaccination campaigns, it is crucial that, along with political will, it is also important to have a decentralized approach with inter-sectoral coordination with different stakeholders such as healthcare workers, community members, and different departments such as the local self-governments, education department, and law and order department. Targets for vaccination coverage are often set by the health department. However, if these targets are jointly discussed and the local self-government is involved in the process of target setting from the very beginning along with the health system, it would lead to ownership of the program and better coordination. Therefore, these lessons learned from COVID-19 vaccination campaigns must not be forgotten and must be applied in future vaccination campaigns.

## Data availability statement

The original contributions presented in the study are included in the article/supplementary material, further inquiries can be directed to the corresponding authors.

## Ethics statement

The studies involving humans were approved by ethics committees of the following ICMR Institutes: ICMR-National AIDS Research Institute, Pune; ICMR-National Institute for Research in Environmental Health, Bhopal; ICMR-Centre for Ageing and Mental Health, Kolkata; ICMR-Regional Medical Research Centre, Gorakhpur; ICMR-National Institute of Epidemiology, Chennai; and ICMR-Regional Medical Research Centre, NE Region, Dibrugarh. The studies were conducted in accordance with the local legislation and institutional requirements. The participants provided their written informed consent to participate in this study.

## Author contributions

SGo: Data curation, Formal analysis, Investigation, Writing – review & editing. VD: Data curation, Formal analysis, Investigation, Writing – review & editing. SD: Data curation, Formal analysis, Investigation, Writing – review & editing. MR: Data curation, Formal analysis, Investigation, Writing – review & editing. MKa: Data curation, Formal analysis, Investigation, Writing – review & editing. DR: Data curation, Formal analysis, Investigation, Writing – review & editing. DB: Data curation, Formal analysis, Investigation, Writing – review & editing. MMa: Data curation, Formal analysis, Investigation, Writing – review & editing. RT: Data curation, Formal analysis, Investigation, Writing – review & editing. JM: Data curation, Formal analysis, Investigation, Writing – review & editing. AM: Data curation, Formal analysis, Investigation, Writing – review & editing. SM: Data curation, Formal analysis, Investigation, Writing – review & editing. MMe: Data curation, Formal analysis, Investigation, Writing – review & editing. NG: Funding acquisition, Project administration, Resources, Supervision, Visualization, Writing – review & editing. SGu: Funding acquisition, Project administration, Supervision, Writing – review & editing. LG: Conceptualization, Data curation, Formal analysis, Funding acquisition, Methodology, Project administration, Resources, Supervision, Validation, Visualization, Writing – original draft. PK: Data curation, Formal analysis, Investigation, Writing – review & editing. TT: Data curation, Formal analysis, Investigation, Writing – review & editing. IS: Data curation, Formal analysis, Investigation, Writing – review & editing. UA: Data curation, Formal analysis, Investigation, Writing – review & editing. JK: Data curation, Formal analysis, Investigation, Writing – review & editing. SP: Data curation, Formal analysis, Investigation, Writing – review & editing. SS: Data curation, Formal analysis, Investigation, Writing – review & editing. KB: Data curation, Formal analysis, Investigation, Writing – review & editing. GY: Data curation, Formal analysis, Investigation, Writing – review & editing. VK: Data curation, Formal analysis, Investigation, Writing – review & editing. PuG: Data curation, Formal analysis, Investigation, Writing – review & editing. KP: Data curation, Formal analysis, Investigation, Writing – review & editing. RK: Data curation, Formal analysis, Investigation, Writing – review & editing. PrG: Data curation, Formal analysis, Investigation, Writing – review & editing. MKh: Data curation, Formal analysis, Investigation, Writing – review & editing. AK: Data curation, Formal analysis, Investigation, Writing – review & editing.

## References

[1] World Health Organization. India rolls out the world’s largest COVID-19 vaccination drive. (2021). Available at: https://www.who.int/india/news/feature-stories/detail/india-rolls-out-the-world-s-largest-covid-19-vaccination-drive (Accessed 14 July 2023).

[2] PIB. National COVID-19 Vaccination Programme Meets Its Goals By Overcoming R&D And Logistical Challenges, Says Economic Survey 2022–2023. (2023) Available at: https://pib.gov.in/pib.gov.in/Pressreleaseshare.aspx?PRID=1894907 (Accessed 29 August 2023).

[3] PIB. National COVID-19 Vaccination Programme Meets Its Goals By Overcoming R&D And Logistical Challenges, Says Economic Survey 2022–2023.(2023) Available at: https://pib.gov.in/pib.gov.in/Pressreleaseshare.aspx?PRID=1894907 (Accessed 14 July 2023).

[4] CoWIN. Dashboard. Available at: https://dashboard.cowin.gov.in/ (2023) (Accessed 14 July 2023).

[5] McCready JL Nichol B Steen M Unsworth J Comparcini D Tomietto M. Understanding the barriers and facilitators of vaccine hesitancy towards the COVID-19 vaccine in healthcare workers and healthcare students worldwide: an umbrella review. PLoS One. (2023) 18:e0280439. doi: 10.1371/journal.pone.0280439, PMID: 37043505 PMC10096263

[6] Ashipala DO Tomas N Costa TG. Barriers and facilitators affecting the uptake of COVID-19 vaccines: a qualitative perspective of frontline nurses in Namibia. SAGE Open Nurs. (2023) 9:237796082311584. doi: 10.1177/23779608231158419 PMC9969425 36861054

[7] PIB. “COVID vaccination Amrit Mahotsava” being implemented as “Jan Abhiyaan” with massive mass mobilization. Available at: https://pib.gov.in/Pressreleaseshare.aspx?PRID=1854692 (2022)

[8] MacDonald NE, SAGE Working Group on Vaccine Hesitancy. Vaccine hesitancy: definition, scope and determinants. Vaccine. (2015) 33:4161–4. doi: 10.1016/j.vaccine.2015.04.036 25896383

[9] Mertens G Lodder P Smeets T Duijndam S. Fear of COVID-19 predicts vaccination willingness 14 months later. J Anxiety Disord. (2022) 88:102574. doi: 10.1016/j.janxdis.2022.102574, PMID: 35512598 PMC9047433

[10] Dhaliwal BK Chandrashekhar R Rattani A Seth R Closser S Jain A. Community perceptions of vaccination among influential stakeholders: qualitative research in rural India. BMC Public Health. (2021) 21:2122. doi: 10.1186/s12889-021-12188-4, PMID: 34794415 PMC8600485

[11] Agarwal SK Naha M. COVID-19 vaccine coverage in India: a district-level analysis. Vaccine. (2023) 11:948. doi: 10.3390/vaccines11050948, PMID: 37243052 PMC10221184

[12] Nikolovski J Koldijk M Weverling GJ Spertus J Turakhia M Saxon L. Factors indicating intention to vaccinate with a COVID-19 vaccine among older U.S. adults. PLoS One. (2021) 16:e0251963. doi: 10.1371/journal.pone.0251963, PMID: 34029345 PMC8143399

[13] WHO. WHO-EURO-2020-1160-40906-55390-eng.pdf. (2021). Available at: https://apps.who.int/iris/bitstream/handle/10665/335820/WHO-EURO-2020-1160-40906-55390-eng.pdf

[14] Dubé E MacDonald NE. COVID-19 vaccine hesitancy. Nat Rev Nephrol. (2022) 18:409–10. doi: 10.1038/s41581-022-00571-2, PMID: 35414006 PMC9004449

[15] World Health Organization. Immunization stress-related response: A manual for program managers and health professionals to prevent, identify and respond to stress-related responses following immunization. (2019) Available at: https://www.who.int/publications-detail-redirect/9789241515948

[16] McMurtry CM. Managing immunization stress-related response: a contributor to sustaining trust in vaccines. Can Commun Dis Rep. (2020) 46:210–8. doi: 10.4745/ccdr.v46i06a10, PMID: 32673376 PMC7343055

[17] Masthi NRR Brahmajosyula A Khamar A Acharya N Bilichod LP Kondath D. Coverage of coronavirus Disease-2019 (COVID-19) booster dose (precautionary) in the adult population: an online Survey. Cureus. (2022) 14:e26912. doi: 10.7759/cureus.26912 35983381 PMC9376214

[18] World Health Organization. SAGE updates COVID-19 vaccination guidance. (2023) Available at: https://www.who.int/news/item/28-03-2023-sage-updates-covid-19-vaccination-guidance (Accessed 1 September 2023).

[19] Bansal P Raj A Mani Shukla D Sunder N. COVID-19 vaccine preferences in India. Vaccine. (2022) 40:2242–6. doi: 10.1016/j.vaccine.2022.02.077, PMID: 35282928 PMC8898737

[20] CoWIN. Available at: https://www.cowin.gov.in/ (2023). (Accessed 14 July 2023).

[21] Kolobova I Nyaku MK Karakusevic A Bridge D Fotheringham I O’Brien M. Vaccine uptake and barriers to vaccination among at-risk adult populations in the US. Hum Vaccin Immunother. (2022) 18:2055422. doi: 10.1080/21645515.2022.2055422 35536017 PMC9248946

[22] PIB. 75 days long ‘COVID vaccination Amrit Mahotsava’ concludes today. (2022) Available at: https://pib.gov.in/pib.gov.in/Pressreleaseshare.aspx?PRID=1863833 (Accessed 14 July 2023).

[23] Kiran T Junaid KP Sharma D Jain L Vij J Satapathy P. Sociodemographic determinants of willingness and extent to pay for COVID-19 vaccine in India. Front Public Health. (2022) 10:880. doi: 10.3389/fpubh.2022.870880, PMID: 35734756 PMC9207713

[24] Duffy C Newing A Górska J. Evaluating the geographical accessibility and equity of COVID-19 vaccination sites in England. Vaccine. (2021) 10:50. doi: 10.3390/vaccines10010050, PMID: 35062711 PMC8781430

[25] Kartoğlu Ü. How can COVID-19 vaccine manufacturers minimize vaccine wastage? Health Affairs Forefront. (2022). Available at: https://www.healthaffairs.org/do/10.1377/forefront.20210824.58595/full/

[26] Zimmerman T Shiroma K Fleischmann KR Xie B Jia C Verma N. Misinformation and COVID-19 vaccine hesitancy. Vaccine. (2023) 41:136–44. doi: 10.1016/j.vaccine.2022.11.014, PMID: 36411132 PMC9659512

